# Cardiovascular magnetic resonance-derived left atrioventricular coupling index and major adverse cardiac events in patients following acute myocardial infarction

**DOI:** 10.1186/s12968-023-00929-w

**Published:** 2023-04-13

**Authors:** Torben Lange, Sören J. Backhaus, Alexander Schulz, Ruben Evertz, Johannes T. Kowallick, Boris Bigalke, Gerd Hasenfuß, Holger Thiele, Thomas Stiermaier, Ingo Eitel, Andreas Schuster

**Affiliations:** 1 Department of Cardiology and Pneumology, University Medical Center Göttingen, Georg-August University, Robert-Koch-Straße 40, 37075 Göttingen, Germany; 2 German Centre for Cardiovascular Research (DZHK), Partner Site Göttingen, Göttingen, Germany; 3 School of Biomedical Engineering and Imaging Sciences, King’s College, London, UK; 4 Institute for Diagnostic and Interventional Radiology, University Medical Center Göttingen, Georg-August University, Göttingen, Germany; 5 Department of Cardiology, Charité Campus Benjamin Franklin, University Medical Center Berlin, Berlin, Germany; 6 Department of Internal Medicine/Cardiology and Leipzig Heart Institute, Heart Center Leipzig at University of Leipzig, Leipzig, Germany; 7 Medical Clinic II (Cardiology/Angiology/Intensive Care Medicine), University Heart Center Lübeck, University Hospital Schleswig-Holstein, Lübeck, Germany; 8 German Center for Cardiovascular Research (DZHK), Partner Site Hamburg/Kiel/Lübeck, Lübeck, Germany

**Keywords:** Cardiovascular magnetic resonance imaging, Acute myocardial infarction, Optimized risk stratification, Left atrioventricular coupling index

## Abstract

**Background:**

Recently, a novel left atrioventricular coupling index (LACI) has been introduced providing prognostic value to predict cardiovascular events beyond common risk factors in patients without cardiovascular disease. Since data on cardiovascular magnetic resonance (CMR)-derived LACI in patients following acute myocardial infarction (AMI) are scarce, we aimed to assess the diagnostic and prognostic implications of LACI in a large AMI patient cohort.

**Methods:**

In total, 1046 patients following AMI were included. After primary percutaneous coronary intervention CMR imaging and subsequent functional analyses were performed. LACI was defined by the ratio of the left atrial end-diastolic volume divided by the left ventricular (LV) end-diastolic volume. Major adverse cardiac events (MACE) including death, reinfarction or heart failure within 12 months after the index event were defined as primary clinical endpoint.

**Results:**

LACI was significantly higher in patients with MACE compared to those without MACE (p < 0.001). Youden Index identified an optimal LACI cut-off at 34.7% to classify patients at high-risk (p < 0.001 on log-rank testing). Greater LACI was associated with MACE on univariate regression modeling (HR 8.1, 95% CI 3.4–14.9, p < 0.001) and after adjusting for baseline confounders and LV ejection fraction (LVEF) on multivariate regression analyses (HR 3.1 95% CI 1.0–9, p = 0.049). Furthermore, LACI assessment enabled further risk stratification in high-risk patients with impaired LV systolic function (LVEF ≤ 35%; p < 0.001 on log-rank testing).

**Conclusion:**

Atrial-ventricular interaction using CMR-derived LACI is a superior measure of outcome beyond LVEF especially in high-risk patients following AMI.

*Trial registration* ClinicalTrials.gov, NCT00712101 and NCT01612312

**Supplementary Information:**

The online version contains supplementary material available at 10.1186/s12968-023-00929-w.

## Introduction

Acute myocardial infarction (AMI) remains a leading cause of morbidity and mortality worldwide although there have been substantial improvements in prognosis over the past decades [1]. Efforts have been directed towards identification of novel non-invasive imaging parameters and indices for improved risk stratification enabling further optimized patient management. In this context, cardiovascular magnetic resonance (CMR) imaging has emerged as a key modality providing comprehensive possibilities for both functional and morphological myocardial assessment in patients following AMI [2, 3]. Besides most commonly used left ventricular (LV) ejection fraction (LVEF), myocardial strain analyses have been proven to possess important and superior prognostic value for optimized risk assessment in AMI patients [4, 5]. However, comprehensive strain analyses can be time-consuming and require additional post-processing software applications [6, 7]. Recently, a new and simple approach of calculating a left atrioventricular coupling index (LACI) that is defined by the ratio between the left atrial (LA) end-diastolic volume (EDV) and the LV EDV has been introduced and demonstrated to be associated with the occurrence of cardiovascular events and to possess an incremental long-term prognostic value over and above traditional clinical risk factors in a large cohort of patients without any cardiovascular disease at study enrollment (Multi-Ethnic Study of Atherosclerosis [MESA study])[8, 9].

Currently, little is known about the importance of left atrioventricular coupling and applicability and prognostic implications of LACI following AMI. Therefore, we aimed to assess the prognostic value of CMR-derived LACI in a large multicenter study of patients with ST-segment elevation myocardial infarction (STEMI) and non-ST-segment elevation myocardial infarction (NSTEMI) treated by primary percutaneous coronary intervention (PCI).

## Methods

### Study population

**Fig. 1:**
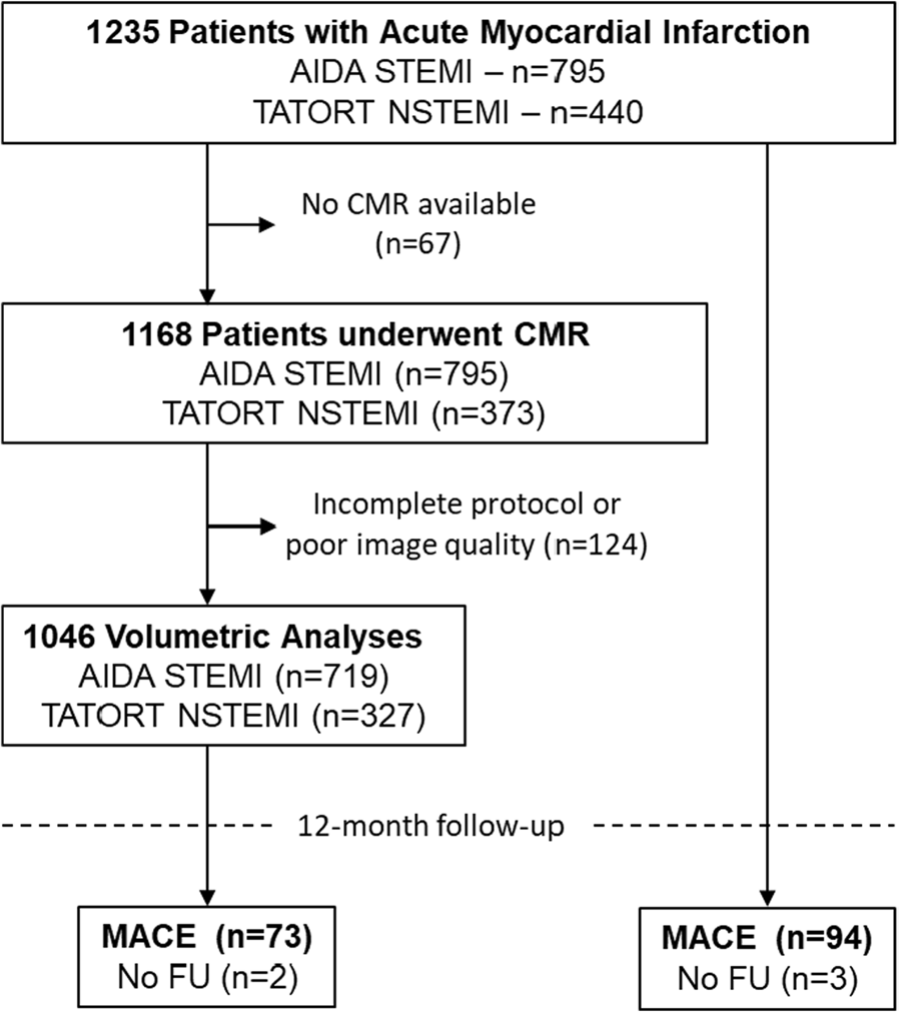
Study flowchart. *AIDA* Abciximab Intracoronary versus Intravenous Drug Application, *CMR* cardiovascular magnetic resonance, *MACE* major adverse cardiac event, *NSTEMI* non–ST segment-elevation myocardial infarction, *STEMI* ST segment-elevation myocardial infarction, *TATORT* Thrombus Aspiration in Thrombus Containing Culprit Lesions

After undergoing primary PCI for AMI, 1168 patients underwent CMR imaging, amongst them 1046 had a complete and analyzable imaging data set and were included to this CMR substudy (Fig. 1). All patients were enrolled within the AIDA-STEMI (Abciximab Intracoronary versus intravenously Drug Application in STEMI) and TATORT-NSTEMI (Thrombus Aspiration in Thrombus Containing Culprit Lesions in NSTEMI) trials. Further information on detailed study protocols and results have been previously reported [10, 11]. All patients gave written informed consent before study participation. All involved local ethical committees approved both studies that complied with the principles of the Helsinki Declaration.

### CMR imaging protocol and analysis

Within 10 days after the index event an identical CMR protocol was applied in all patients. The protocol was performed on 1.5 or 3 T CMR scanners at the respective study sites including balanced steady-state free precession sequences (bSSFP) of long-axis 2- and 4-chamber views as well as short axis (SAx) stacks. Typical bSSFP sequence parameters were as follows: TR 3.2 ms, TE 1.2 ms, flip angle 60°, 8 mm slice thickness in SAx. More detailed information on CMR scan protocols have been published previously [10, 11]. Likewise, typical contraindications to CMR applied to this study as listed elsewhere [11]. Manual post-processing was performed in cine bSSFP images using dedicated evaluation software (cmr^42^ Circle Cardiovascular Imaging Inc., Calgary, Alberta, Canada). To assess LV global longitudinal strain (GLS), LV epi- and endocardial borders were manually tracked in 2- and 4-chamber [12]. Likewise, LA total strain was obtained from 2- and 4-chamber images by manually delineating LA endocardial borders [13]. After manual delineation of the myocardial borders at end-diastole a semi-automated tracking algorithm was applied for tracing the contours throughout the cardiac cycle. Subsequently, visual reviews of the semi-automatically tracked contours were performed and in case of insufficient border tracking, manual adjustments were made followed by a subsequent reapplication of the algorithm. All peak strain measurements are presented in percent and based on an average of three repeated and independent tracking repetitions [14]. 

### Left atrioventricular coupling index

LACI was calculated as a ratio between CMR-derived LA EDV and LV EDV as previously defined [9]. LA and LV volumes were measured in the same end-diastolic phase.

**Fig. 2:**
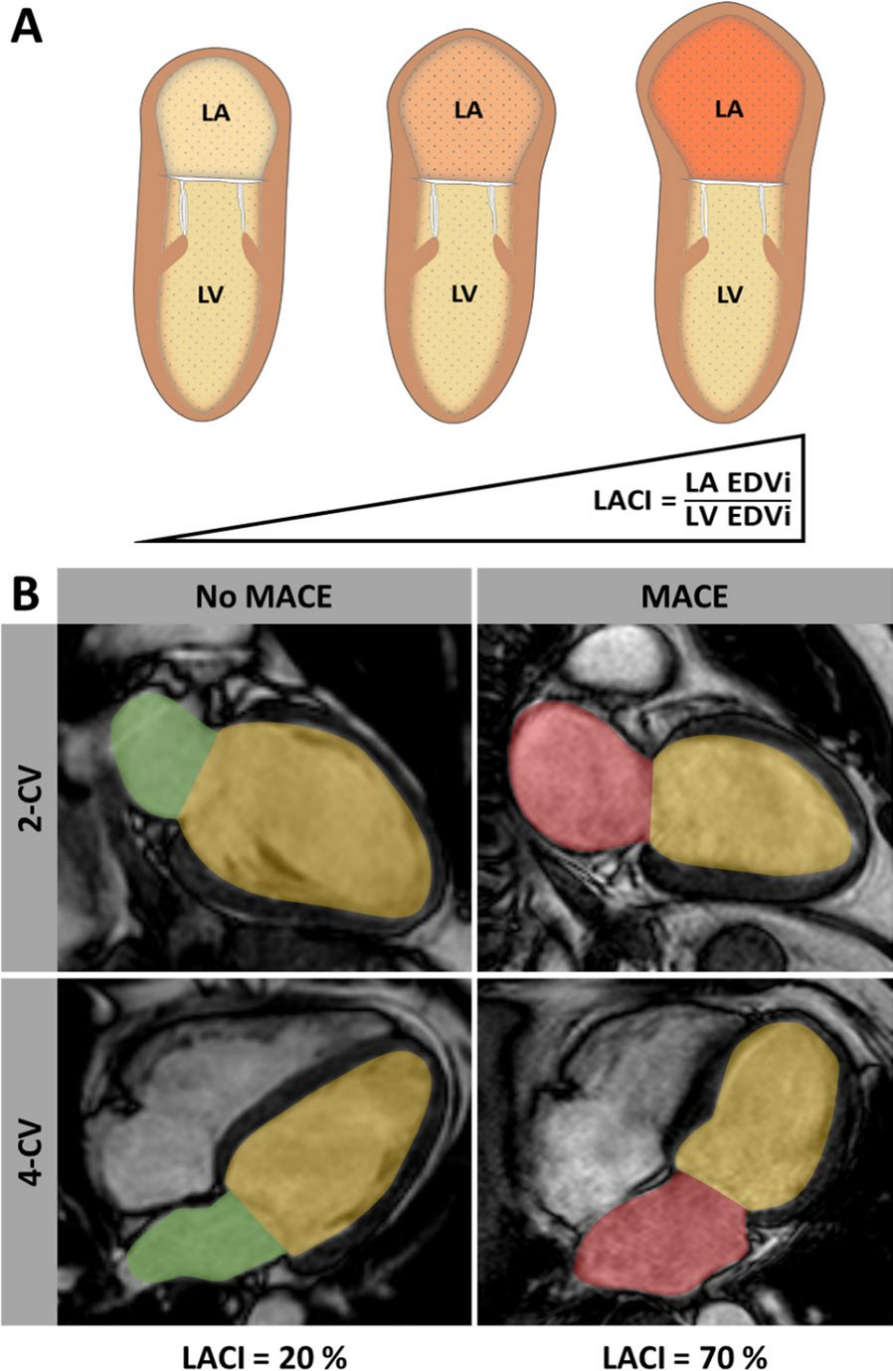
Left atrioventricular coupling index (LACI). **A** Schematic illustration of the left atrioventricular coupling index (LACI), which is defined as a ratio between left atrial (LA) and left ventricular (LV) end-diastolic volume index (EDVI). Proportional enlargement of LA EDVI leads to an increase of LACI. **B** LACI indicating differences of LA (red/green) and LV (yellow) volume ratio in 2- and 4-chamber views of a patient with and without a major adverse clinical event (MACE), respectively

LACI value is expressed as a percentage. Consequently, a higher LACI reflects a greater imbalance between the LA and LV volumes at ventricular end-diastole suggesting greater impairment of left atrioventricular coupling (Fig. 2A). In addition, a LACI measured in LA and LV end-systole (LACI ES) was calculated.

### Clinical endpoints and outcome

All-cause mortality, reinfarction or heart failure associated with rehospitalization within the first year after AMI were counted as major adverse cardiac event (MACE), which was defined as the primary clinical endpoint of this study. In case of multiple occurrences of MACE within one patient, a prioritization was made as follows: death > reinfarction > heart failure. Furthermore, each patient could only account for one MACE.

### Statistical analyses

Categorical parameters are presented in absolute numbers and percentages while continuous parameters were tested for normal distribution using Shapiro–Wilk test and are reported as mean with interquartile range (IQR). For the assessment of correlations, the spearman´s rank correlation coefficient was used. Non-parametric Mann–Whitney U test was used for comparisons of continuous data sets. An optimal dichotomization cut-off value for LACI was determined using Youden’s Index. Moreover, patients with a LVEF ≤ 35% were classified as a high-risk group according to established clinical practice. To analyze occurrence of clinical endpoints the Kaplan–Meier method was applied and log-rank testing was used for assessing differences between groups. Cox proportional regression models were applied for the calculation of univariate hazard ratios (HRs) in context of MACE and mortality evaluation. Only variables with a p-value < 0.05 were included in further multivariable regression calculations. A stepwise approach with thresholds of 0.05 and 0.1 for p-values to keep or remove variables respectively was used in multivariate calculations. Due to significant correlations of LACI and LA or LV volumes only one of these parameters was included in multivariate regression models in each case. In addition, the improvement in discrimination by adding LACI to a risk prediction model containing LVEF was assessed using net reclassification improvement (NRI) and integrated discrimination improvement (IDI) measurements [15]. Reproducibility testing was performed on 100 randomly selected cases (50 STEMI and 50 NSTEMI). Provided p-values are two-sided with an alpha level < 0.05 considered statistically significant. For all statistical calculations SPSS (version 28, Statistical Package for the Social Sciences, International Business Machines, Armonk, New York, USA) and Microsoft Excel (Microsoft, Redmond, Washington, USA) were used.

## Results

### Study population

A total of 1168 patients (795 STEMI, 373 NSTEMI) underwent initial CMR imaging in median 3 days (IQR 2–4 days) after the acute index event. Due to incomplete scan protocol or insufficient image quality 1046 patients (719 STEMI, 325 NSTEMI) entered final functional analyses for this study; 2 patients were lost to follow-up and not further included in outcome calculations (Fig. 1). Detailed baseline characteristics of the study population are presented in Table 1. The median age of the study population was 64 years (IQR 53–73 years) with mainly male patients (75%) being included. Typical cardiovascular risk factors were more frequent in patients with MACE than in those without MACE (p = 0.01 for hypertension and p = 0.03 for diabetes) while patients with MACE were less often active smokers than those without MACE (p = 0.047).

**Table 1:** Baseline characteristics

Variables	All patients (n = 1046)	MACE (n = 73)	No MACE (n = 971)	p-value
Age	64 (53–73)	71 Y	63 (52–72)	** < 0.001**
Sex (male)	784/1046 (75)	49/73 (67.1)	734/971 (75.6)	**0.14**
*Cardiovascular risk factors*				
Active smoking	421/967 (40.2)	21/66 (28.8)	399/899 (41.1)	**0.047**
Hypertension	742/1044 (70.9)	61/73 (83.6)	679/969 (69.9)	**0.01**
Hyperlipoproteinemia	394/1041 (37.7)	26/73 (35.6)	367/966 (37.8)	0.7
Diabetes	246/1044 (23.7)	25/73 (34.2)	220/969 (22.7)	**0.03**
Body mass index (kg/m^2^)	27.5 (24.9–30.4)	27.6 (25.4–31.1)	27.4 (24.9–30.2)	0.49
Previous myocardial infarction	75/1044 (7.2)	5/73 (6.8)	69/969 (7.1)	0.93
Previous PCI	90/1045 (8.6)	5/73 (6.8)	84/970 (8.7)	0.59
Previous CABG	19/1046 (1.8)	2/73 (2.7)	17/970 (1.8)	0.54
ST-segment elevation	719/1046 (68.7)	49/73 (67.1)	670/971 (69)	0.74
Systolic blood pressure (mmHg)	133 (118–150)	133 (100–150)	133 (120–150)	0.23
Diastolic blood pressure (mmHg)	80 (70–89)	78 (66–85)	80 (70–89)	**0.08**
Heart rate (bpm)	76 (67–86)	80 (70–95)	76 (66–86)	**0.001**
Time symptoms to balloon* (min)	180 (109–317)	194 (115–381)	180 (108–310)	0.22
Door-to-balloon time* (min)	30 (22–42)	28 (23–40)	30 (22–42)	0.45
*Killip class on admission*				** < 0.001**
1	925/1046 (88.4)	48/73 (65.8)	875/971 (90.1)	
2	83/1046 (7.9)	16/73 (21.9)	67/971 (6.9)	
3	22/1046 (2.1)	5/73 (6.8)	17/971 (1.8)	
4	16/1046 (1.5)	4/73 (5.5)	12/971 (1.2)	
*Diseased vessels*				**0.008**
1	524/1046 (50.1)	27/73 (37)	496/971 (51.1)	
2	315/1046 (30.1)	22/73 (30.1)	293/971 (30.2)	
3	207/1046 (19.8)	24/73 (32.9)	182/971 (18.7)	
*Affected artery*				0.27
Left anterior descending	430/1046 (41.1)	38/73 (52.1)	392/971 (40.4)	
Left circumflex	217/1046 (20.7)	14/73 (19.2)	201/971 (20.7)	
Left main	4/1046 (0.4)	0/73 (0)	4/971 (0.4)	
Right	388/1046 (37.1)	20/73 (27.4)	368/971 (37.9)	
Bypass graft	7/1046 (0.7)	1/73 (1.4)	6/971 (0.6)	
*TIMI flow grade before PCI*				0.62
0	524/1046 (50.1)	41/73 56.2)	482/971 (49.6)	
1	112/1046 (10.7)	5/73 (6.8)	107/971 (11)	
2	219/1046 (20.9)	14/73 (19.2)	204/971 (21)	
3	191/1046 (18.3)	13/73 (17.8)	178/971 (18.3)	
Stent implanted	1022/1046 (97.7)	71/73 (97.3)	949/971 (97.7)	0.62
*TIMI flow grade after PCI*				0.11
0	20/1046 (1.9)	1/73 (1.4)	19/971 (2)	
1	22/1046 (2.1)	4/73 (5.5)	18/971 (1.9)	
2	78/1046 (7.5)	8/73 (11)	70/971 (7.2)	
3	926/1046 (88.5)	60/73 (82.2)	864/971 (89)	
*Medication*				
Aspirin	1044/1046 (99.8)	73/73 (100)	969/971 (99.8)	0.7
Clopidogrel/Prasugrel/Ticagrelor	741/1045 (70.8)	73/73 (100)	971/971 (100)	
Betablocker	1000/1046 (95.6)	71/73 (97.3)	927/970 (95.5)	0.7
ACE-inhibitor/AT-1 antagonist	961/1045 (91.9)	69/73 (94.5)	891/970 (91.8)	0.42
Aldosterone antagonist	137/1046 (13.1)	24/73 (32.9)	113/970 (11.6)	** < 0.001**
Statin	1007/1045 (96.3)	71/73 (97.3)	934/970 (96.2)	0.67
Time to CMR (days)	3 (2–4)	3 (2–4)	3 (2–4)	0.05

Data are presented as n/N (%) or median (interquartile range). Two patients were lost to follow-up regarding MACE. For comparison of patients with MACE and no MACE p-values were calculated, bold numbers indicate a statistically significant difference. Mann–Whitney U test was used for testing continuous variables, categorical variables were tested using chi square test

ACE: angiotensin converting enzyme; CABG: coronary artery bypass graft; MACE: major adverse cardiac event; PCI: percutaneous coronary intervention, TIMI: thrombolysis in myocardial infarction

### CMR imaging and left atrioventricular coupling index

The median LACI of the study population was 21.4% (15.9%–29.2%). There was no difference of LACI between patients with STEMI or NSTEMI (p = 0.12). Underlying parameters of LA EDV and LV EDV significantly correlated (r = 0.4, p < 0.001). There was no correlation of LACI with LV functional parameters (GLS: r =—0.001, p = 0.96; LVEF: r = -0.03, p = 0.32) but with LA functional parameters (LA total strain r = -0.58, p < 0.001, LA emptying fraction (LAEF): r = -0.67, p < 0.001). A detailed overview of CMR parameters is displayed in Table 2. Reproducibility of LACI was excellent according to intraclass correlation coefficient (ICC) (0.98 [0.98–0.99]) with a coefficient of variation of 4.1%.

**Table 2:** Cardiac magnetic resonance results

	All patients (n = 1046)	MACE (n = 73)	No MACE (n = 971)	p-value
LACI (%)	21.4 (15.9–29.2)	28.0 (19.5–42.1)	21.0 (15.7–28.4)	**< 0.001**
LVEF (%)	50.6 (43.5–57.5)	41.2 (33.1–52.4)	50.9 (44.3–57.6)	** < 0.001**
LV EDVI (mL/m^2^)	73.3 (62.0–85.7)	75.0 (66.5–86.6)	73.0 (62.1–85.6)	0.23
LV ESVI (mL/m^2^)	35.6 (27.8–45.9)	44.8 (31.4–54.0)	35.2 (27.6–45.4)	** < 0.001**
LV SVI (mL/m^2^)	36.2 (30.6–42.3)	33.1 (25.2–38.0)	36.6 (30.9–42.5)	** < 0.001**
LA EF (%)	53.2 (46.3–59.2)	44.2 (35.2–52.0)	53.7 (47.0–59.5)	** < 0.001**
LA EDVI (mL/m^2^)	15.7 (11.6–21.9)	21.0 (14.1–34.6)	15.3 (11.5–21.3)	** < 0.001**
LA ESVI (mL/m^2^)	35.0 (26.5–44.4)	40.5 (28.0–53.6)	34.5 (26.5–43.6)	**0.004**
LV GLS (%)	− 16.4 (− 12.4–− 20.1)	− 11.6 (− 8.3–− 17.1)	− 16.7 (− 12.9–− 20.4)	** < 0.001**
LA Reservoir strain (%)	20.9 (16.2–25.7)	16.2 (11.6–21.3)	21.2 (16.7–26.1)	** < 0.001**

Values are displayed as median (interquartile range). Two patients were lost to follow-up regarding MACE. P-values were calculated for the comparison between patients with and without MACE using the Mann–Whitney U test. Numbers in bold indicate a statistical significance in difference

EDVI: end-diastolic volume index; ESVI: end-systolic volume index; GLS: global longitudinal strain, LA: left atrial, LACI: left atrioventricular coupling index, LVEF: left ventricular ejection fraction

### Prognostic value of Left atrioventricular coupling index

**Fig. 3:**
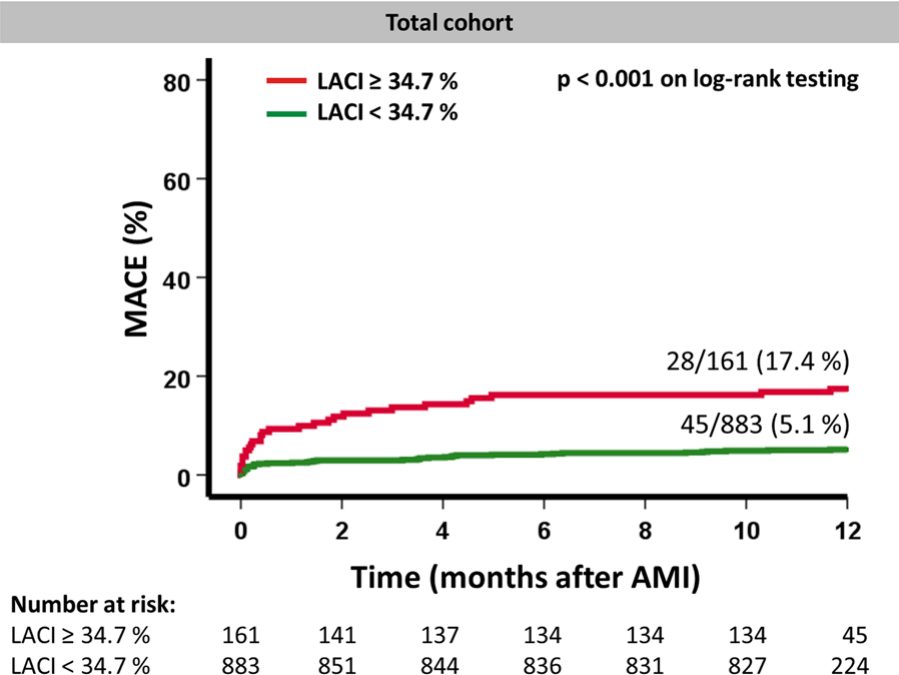
Kaplan–Meier curves for survival analyses. Left atrioventricular coupling index (LACI) and survival after acute myocardial infarction (AMI). Incidence of MACE (major adverse cardiac events) according to high and low LACI classified according to Youden Index

Amongst the 1046 study patients that entered final outcome analyses, 73 MACE were documented during 1-year follow-up (death = 34, reinfarction = 18, heart failure = 21). In patients with MACE LACI was significantly higher compared to those patients without MACE (28.0% [IQR 19.5–42.1] vs. 21.0% [IQR 15.7–28.4], p < 0.001) (Fig. 2). In the overall cohort Youden Index identified an optimal LACI cut-off of 34.7% to best classify patients into low- and high-risk groups according to LACI (p < 0.001 on log-rank testing) (Fig. 3).

In univariate regression analyses a greater LACI was significantly associated with MACE (HR 8.1, 95% CI 3.4c14.9, p < 0.001) and remained significantly associated with MACE after adjusting for baseline confounders and LVEF on multivariate cox regression analyses (HR 3.1 95% CI 1.0–9.4, p = 0.049) (Table 3). When LV GLS or LA total strain were included to the multivariate cox regression model, LACI was not associated with MACE anymore. Significant associations with MACE in regression analyses were also documented when dividing the study population into STEMI and NSTEMI subgroups (HR 8.4 95% CI 3.0–23.8, p < 0.001 in STEMI patients and HR 7.2 95% CI 1.36–38.3, p = 0.02 in NSTEMI patients). Similarly, LACI was independently associated with MACE both in female and male patients (female: HR 6.9 (95% CI 1.9–25.4), p = 0.004; male: HR 8.0 (95% CI 2.2–29.6), p = 0.002). For LACI ES no significant association with MACE was documented (HR 0.93 [95% CI 0.57–1.52], p = 0.78).

**Table 3:** Univariate and multivariate Cox regression analysis for prediction of MACE

Variables	Univariate Hazard ratio (CI)	p-value	Multivariate Hazard ratio (CI)	p-value
Age	1.04 (1.02–1.06)	** < 0.001**	1.02 (0.99–1.04)	0.12
Hypertension	0.47 (0.25–0.88)	**0.017**	0.67 (0.34–1.33)	0.25
Diabetes	0.58 (0.36–0.94)	**0.026**	0.79 (0.47–1.34)	0.38
Heart rate (bpm)	1.03 (1.01–1.04)	** < 0.001**	1.01 (1.0–1.03)	0.051
Killip class on admission	2.02 (1.59- 2.56)	** < 0.001**	1.5 (1.1–2.0)	**0.006**
Number of diseased vessels	1.5 (1.1–2.0)	**0.004**	1.24 (0.91–1.69)	0.18
Infarct size (ml)	1.0 (1.0–1.02)	**0.015**	0.99 (0.99–1.01)	0.84
LVEF (%)	0.94 (0.92–0.96)	** < 0.001**	0.96 (0.93–0.98)	** < 0.001**
LACI (%) ^†^	8.1 (3.4–19.7)	** < 0.001**	3.1 (1.0–9.4)	**0.049**
LV EDVI (mL/m^2^)^†^	1.01 (1.0–1.02)	0.07	1.0 (0.98–1.01)	0.99
LV ESVI (mL/m^2^)^†^	1.03 (1.02–1.04)	** < 0.001**	1.0 (0.98–1.02)	0.74
GLS (%) *	1.14 (1.1–1.2)	** < 0.001**		
LA EDVI (mL/m^2^)^†^	1.01 (1.0–1.02)	** < 0.001**	1.02 (1.0–1.03)	**0.02**
LA ESVI (mL/m^2^)^†^	1.02 (1.01–1.03)	** < 0.001**	1.01 (0.99–1.03)	0.08
LA Total strain (%) *	0.91 (0.87–0.94)	** < 0.001**		

^†^ Due to high correlations of LA and LV volumes with LACI, only LACI or sole LA or LV volumes were included to multivariate regression models

* LV GLS and LA total strain were not included to the presented multivariate regression models due to their outperforming associations with MACE compared to LACI and LV ejection fraction (LVEF)

EDVI: end-diastolic volume index, ESVI: end-systolic volume index, GLS: global longitudinal strain, LA: left atrial, LACI: left atrioventricular coupling index, LVEF: left ventricular ejection fraction

In subanalyses regarding each clinical endpoint separately, LACI showed significant associations with MACE in death (HR 10.8 [95% CI 3.1–37.3], p < 0.001) and heart failure (HR 10.3 [95% CI 3.1–33.8], p < 0.001) but not in those with reinfarction (HR 2.9 [95% CI 0.38–21.2], p = 0.31).

**Fig. 4:**
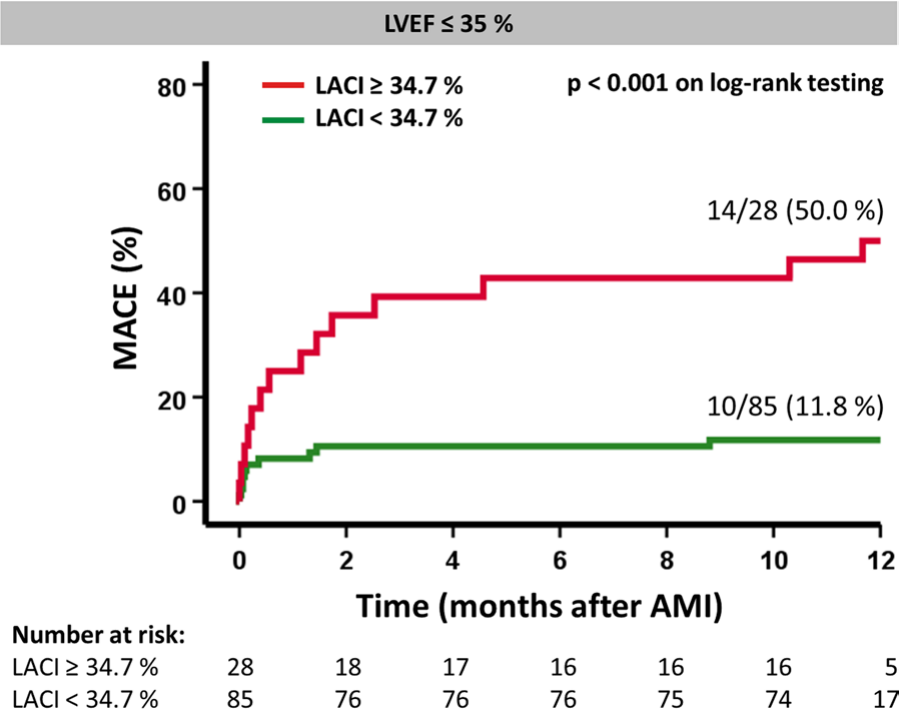
Kaplan–Meier curves for survival analyses in subgroup of high-risk patients. Left atrioventricular coupling index (LACI) and survival in high-risk patients according to left ventricular ejection fraction (LVEF) after acute myocardial infarction (AMI). Incidence of MACE (major adverse cardiac events) according to high and low LACI classified according to Youden Index

Assessing the prognostic improvement in discrimination of different models, the addition of LACI to LVEF resulted in significant improvement using IDI (p = 0.013) and continuous NRI (p = 0.02). Furthermore, Kaplan–Meier plots and using optimal cut-off values identified by Youden Index LACI assessment enabled further risk stratification in high-risk patients according to reduced LVEF ≤ 35% (p < 0.001 on log-rank testing) (Fig. 4), while this was not possible by sole LA or LV volume assessment (p = 0.78 for LV EDV index (EDVI) and p = 0.09 for LA EDVI). Likewise, additional risk stratification in high-risk patients according to reduced LVEF ≤ 35% was possible both in female (p = 0.007 on log-rank testing) and male patients (p < 0.001 on log-rank testing). Dichotomization into low- and high-risk groups amongst patients with LVEF > 35% was feasible applying a threshold of 18.7% (p = 0.001 on log-rank testing) (Additional file 1: Fig. S1).

## Discussion

The aim of this work was to assess a novel CMR-derived LACI and confirm its prognostic value in a large cohort of patients with AMI.

Recently, LACI was defined and proven to possess prognostic value as well as to improve risk classifications in the multi-ethnic population of the MESA study. Likewise, the results of our study demonstrated prognostic implications of this novel index in a large AMI patient cohort. Especially in patients at high-risk according to reduced LVEF LACI evaluation enabled further risk stratification and therefore could optimize clinical patient management.

While commonly used volumetric analyses largely disregard the atrioventricular interplay, a combination of simultaneous LA and LV EDV measurements expressed by LACI can be suggested to allow evaluation of the holistic cardiac performance more accurately.

Currently, many guideline recommendations and clinical decisions (e.g. for ICD device therapy) are mainly based on a LVEF cut-off of 35% which alone may not be sufficient for this purpose [16]. Consequently, there is a special interest to further improve and facilitate the identification of patients at higher risk [17]. Similar to the findings of Pezel et al. [8, 9], we demonstrated high associations of LACI with MACE in our study cohort. Importantly, LACI was shown to have a better prognostic value than individual LA or LV parameters measured separately in high-risk patients according to commonly used LVEF cut-off of 35%. In addition, a lower LACI almost eliminates the risk of MACE occurrence and provides further risk stratification in patients at relatively little risk according to LVEF > 35%. Conversely, the higher cut-off value for the overall cohort best enabled risk classification in patients at higher jeopardy (LVEF ≤ 35%).

Of note, the identified cut-off value of the current study was considerably higher than reported in previous literature [9]. However, since preceding works mainly assessed populations without cardiovascular diseases, the identified value might be characteristic for post AMI patients and may further vary amongst other cardiovascular pathologies. The current findings therefore confirm and emphasize the important value of LACI assessment of recent studies and demonstrate their prognostic potential in an AMI cohort. Importantly, beyond previously identified associations of an increasing LACI with cardiovascular risk factors, markers of myocardial fibrosis and markers of heart failure [18] the reported results are the first suggesting an additional prognostic benefit of LACI in patients with significantly reduced LVEF. Furthermore, with sex-specific pathophysiological features gaining increasing attention in different diseases [19], very recently an association between LACI and sex hormone levels has been suggested influencing left atrioventricular coupling [20]. In our study, LACI was associated with MACE and enabled additional identification of high-risk patients equally both in female and male patients suggesting uniform, accurate and gender independent risk assessment following AMI.

Since a greater LACI suggests an increased mismatch with a disproportionately enlarged LA in relation to LV, this index represents a simple approach to unmask several pathophysiological mechanisms of cardiac performance. LA size is considered as an appropriate barometer of LV filling pressure [21]*.* Furthermore, especially during end-diastole the LA is directly exposed to LV pressure making it an appropriate surrogate parameter for LV diastolic function [22]. In this context, LACI has been shown to identify heart failure patients with preserved ejection fraction [23] and besides indicating LV diastolic dysfunction to a certain extent [24], both LA EDV and end-systolic volume (ESV) were demonstrated to possess important prognostic implications in patients following AMI with LA EDV being superior to ESV [22, 25]. Whether these alterations of LA volumetric geometry occur merely in response to raised LV pressures or whether they also indicate intrinsic atrial processes leading to subsequent heart failure and atrial cardiomyopathy cannot be fully answered [26]. However, it is interesting, that although there was no significant difference of LV EDV between patients suffering MACE and those without MACE during 1-year follow-up, LA volumes differed significantly and, therefore, divergent atrial responses and compensation capabilities can be assumed. Nevertheless, sole LA volume assessment did not allow further risk stratification amongst high-risk patients with a reduced LVEF ≤ 35% in our work. Recent studies identified various cardiovascular risk factors influencing LACI and its changes over time. Furthermore, LACI was demonstrated to be associated with myocardial fibrosis [18] and to be superior for the prediction of new-onset atrial fibrillation compared to conventional risk factors and LA parameters [27, 28]. Consequently, capturing myocardial volume distribution and relation between both LA and LV seems reasonable from a physiological point of view and also regarding improved prognostic stratification by combining prognostically powerful volumetric parameters.

It is noteworthy, that the alternatively calculated LACI ES showed no association with MACE in our study cohort, which, however, is in line with previous findings of the MESA study [9] and underlines reports of other studies demonstrating LA EDV as superior parameter for reflecting LV filling pressure and for prognosticating clinical outcomes compared to LA ESV [29, 30].

Similar to the calculation of LACI, first imaging studies already applied and implemented functional deformation assessments to evaluate the atrioventricular interplay for optimized diagnostic and prognostic purposes [31, 32]. Especially the combination of CMR-derived LA and LV strain assessments revealed important insights to interrelated atrial dysfunction and ventricular systolic compensation mechanisms [19]. It is important to mention, that both ventricular and atrial strain assessments have been shown to provide incremental prognostic value in patients following AMI outperforming myocardial volumetric analyses in several studies [33, 34]. Besides the fact that comprehensive LA and LV strain assessments reflect cardiac performance over the whole cardiac cycle on both global and regional levels, it is also known that strain alterations precede myocardial geometric/ volumetric changes enabling earlier and more precise diagnosis of myocardial performance deterioration [35]. In this context, previously LA total strain has even been shown to identify patients with diastolic heart failure more accurately than invasive pressure measurements [36, 37] underlining the decisive and outclassing nature of strain evaluations compared to volumetric evaluations. 

However, although LACI assessment per se cannot replace or achieve a similar level of prognostic power like LA or LV strain parameters can provide, it is important to consider its role as an index parameter measuring atrioventricular proportions and a growing volumetric mismatch could indicate an aspect of cardiac functional failure that might not be adequately captured by (isolated) LA and/or LV strain deterioration. Consequently, not only amongst LA and LV volumetric analyses but also in addition to strain measurements LACI calculation could give clinicians additional prognostic data to identify patients at higher risk for MACE.

Of note, deformation imaging relies on accurate data acquisition and compared to LACI calculation time-consuming post-processing [38]. Furthermore, simple implementation of LACI in clinical routine is possible without any further post-processing work steps or software applications required making LACI an attractive software independent as well as cost- and time-saving imaging parameter with important diagnostic and prognostic implications. Due to the dimensionless nature of this index it might be even easily transferrable to commonly used echocardiographic volumetric assessments and could be directly comparable between different imaging modalities which has to be validated by future studies. However, a potential influence of methodologic variations on LA and LV volumes by using multislice approaches or biplane techniques should be considered. To obtain best comparable values, the application of a consistent and reproducible method in clinical trials or practice is highly desirable [39, 40]. Against this background, future 3-dimensional and/or artificial intelligence based post-processing software algorithms might further standardize as well as improve volumetric analyses and could also automatically incorporate LACI calculations for the development of new risk prediction models [41, 42].

### Limitations

Our study has several limitations. CMR imaging was performed at several study sites using different CMR-vendors. However, all centers followed an identical study protocol and centralized image post-processing was performed in an experienced and blinded core laboratory. It is noteworthy, that an optimal timepoint for CMR imaging in patients following AMI is not known and, therefore, it cannot be excluded that further changes of atrioventricular coupling might occur at later stages after AMI. Due to contraindications and length of an CMR scanning procedure only stable and preselected patients were included in this study, which might lead to a selection bias with a lower event rate. Nevertheless, the study demonstrated significant associations of LACI with MACE which could be even more pronounced in the presence of more MACE.

## Conclusion

This study confirmed previous findings of calculating a CMR-derived LACI for optimizing risk stratification of cardiovascular events and demonstrated its usability and prognostic value in patients following AMI. LACI was significantly associated with MACE in AMI patients at 1-year follow-up and enabled better risk stratification than sole LA or LV volume analyses especially in high-risk patients according to reduced LVEF. Simple calculation of LACI adds a further valuable parameter for the identification of patients at higher risk for MACE and can be easily implemented in clinical routine.

## Supplementary Information

**Additional file 1: Figure S1.** Kaplan–Meier curves for survival analyses in subgroup of low-risk patients. Left atrioventricular coupling index (LACI) and survival in low-risk patients according to left ventricular ejection fraction (LVEF) after acute myocardial infarction. Incidence of MACE (major adverse cardiac events) according to high and low LACI classified according to Youden Index.

## Data Availability

Regarding data availability, we confirm that all relevant data are within the paper and all data underlying the findings are fully available without restriction and can be accessed at the University Medical Centre Göttingen by researchers who meet the criteria for access to confidential data.

## Abbreviations

AMI: Acute myocardial infarction
bSSFP: Balanced steady state free precession
CMR: Cardiovascular magnetic resonance
EDV: End-diastolic volume
EDVI: End-diastolic volume index
ES: End-systole
ESV: End-systolic volume
ESVI: End-systolic volume index
GLS: Global longitudinal strain
IDI: Integrated discrimination improvement
LA: Left atrium/left atrial
LACI: Left atrial coupling index
LAEF: Left atrial emptying fraction
LV: Left ventricle/left ventricular
LVEF: Left ventricular ejection fraction
MACE: Major adverse cardiovascular event
NRI: Net reclassification improvement
NSTEMI: Non-ST segment elevation myocardial infarction
PCI: Percutaneous coronary intervention
SAx: Short axis
STEMI: ST segment elevation myocardial infarciton
