# Supplementary material for: Cardiovascular magnetic resonance-derived left atrioventricular coupling index and major adverse cardiac events in patients following acute myocardial infarction
Source: J Cardiovasc Magn Reson. 2023 Apr 13;25:24. doi: 10.1186/s12968-023-00929-w (PMC10099819; doi:10.1186/s12968-023-00929-w)
Supplement: Supplementary file 1 — Additional file 1: Figure S1. Kaplan–Meier curves for survival analyses in subgroup of low-risk patients. Left atrioventricular coupling index (LACI) and survival in low-risk patients according to left ventricular ejection fraction (LVEF) after acute myocardial infarction. Incidence of MACE (major adverse cardiac events) according to high and low LACI classified according to Youden Index. [file 12968_2023_929_MOESM1_ESM.docx]

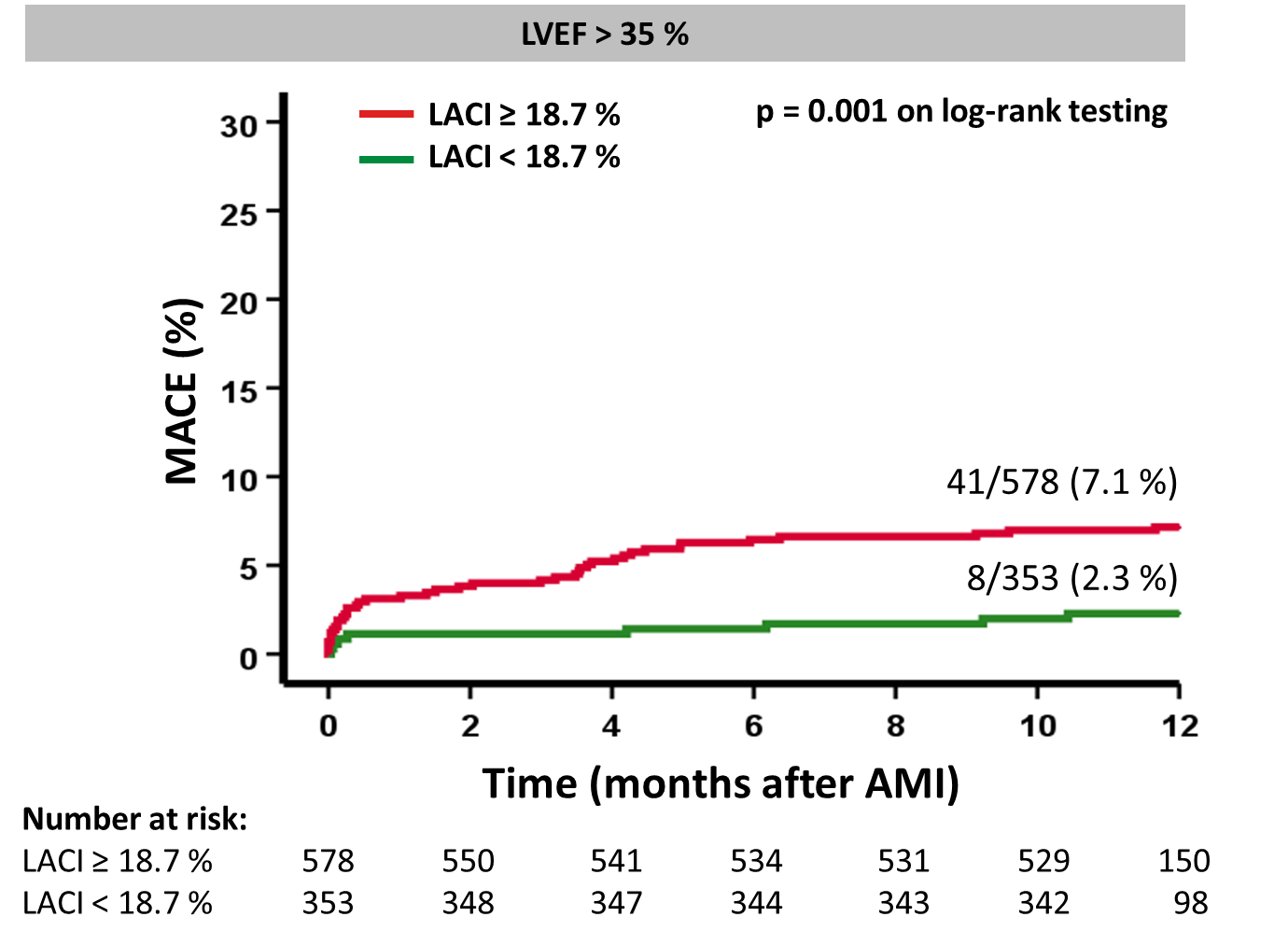


**Figure S1 Kaplan-Meier curves for survival analyses in subgroup of low-risk patients**Left atrioventricular coupling index (LACI) and survival in low-risk patients according to left ventricular ejection fraction (LVEF) after acute myocardial infarction. Incidence of MACE (major adverse cardiac events) according to high and low LACI classified according to Youden Index.
